# Raddeanin A suppresses breast cancer-associated osteolysis through inhibiting osteoclasts and breast cancer cells

**DOI:** 10.1038/s41419-018-0417-0

**Published:** 2018-03-07

**Authors:** Qiang Wang, Jian Mo, Chenchen Zhao, Kangmao Huang, Mingxuan Feng, Wenxin He, Jiying Wang, Shuai Chen, Zi’ang Xie, Jianjun Ma, Shunwu Fan

**Affiliations:** 1 Department of Orthopaedic Surgery, Sir Run Run Shaw Hospital, Medical College of Zhejiang University, Sir Run Run Shaw Institute of Clinical Medicine of Zhejiang University, 3# East Qingchun Road, Hangzhou, Zhejiang Province 310016 China; 2 Department of Orthopedic Surgery, the Second Affiliated Hospital, Medical College of Zhejiang University, 88#Jiefang Road, Hangzhou, Zhejiang Province 310016 China; 3 Orthopaedic Department, Taizhou Central Hospital, Affiliate Hospital Of Taizhou University, Taizhou, Zhejiang Province 318000 China

## Abstract

Bone metastasis is a severe complication of advanced breast cancer, resulting in osteolysis and increased mortality in patients. Raddeanin A (RA), isolated from traditional Chinese herbs, is an oleanane-type triterpenoid saponin with anticancer potential. In this study, we investigated the effects of RA in breast cancer-induced osteolysis and elucidated the possible mechanisms involved in this process. We first verified that RA could suppress osteoclast formation and bone resorption in vitro. Next, we confirmed that RA suppressed Ti-particle-induced osteolysis in a mouse calvarial model, possibly through inhibition of the SRC/AKT signaling pathway. A breast cancer-induced osteolysis mouse model further revealed the positive protective effects of RA by micro-computed tomography and histology. Finally, we demonstrated that RA inhibited invasion and AKT/mammalian target of rapamycin signaling and induced apoptosis in MDA-MB-231 cells. These results indicate that RA is an effective inhibitor of breast cancer-induced osteolysis.

## Introduction

Anemone raddeana Regel has been widely used to treat cancer, rheumatism, and neuralgia^1–3^. This traditional Chinese medicinal herb belongs to the Ranunculaceae family and exhibits antitumor efficacy, anti-inflammatory efficacy, and analgesic activity^4^. Raddeanin A (RA), an oleanane-type triterpenoid saponin, has been shown to be the main bioactive constituent of Anemone raddeana Regel^4–6^. Recent studies have demonstrated that RA can prevent proliferation, induce apoptosis, and inhibit invasion in various human tumor cells, including gastric cancer cells, hepatocellular carcinoma cells, and non-small-cell lung carcinoma cells^6–8^. The mechanisms through which RA exerts these effects may be attributed to its ability to inhibit angiogenesis by preventing the phosphorylation of vascular endothelial growth factor receptor 2 and associated protein kinases, including phospholipase C γ1, Janus kinase 2, focal adhesion kinase, Src, and AKT^9^. Further research has indicated that RA can also induce apoptosis and autophagy in SGC-7901 cells^10^. Therefore, RA may be a promising agent with broad antitumor effects.

Breast cancer is the most common cancer in women worldwide and is related to a high frequency of bone metastasis. A previous report demonstrated that bone metastasis occurs in 70% of patients who died from prostate cancer or breast cancer^11^. The mechanism of bone metastasis, sometimes referred to as the “vicious cycle,” is complex and involves interactions among metastatic breast cancer cells, osteoblasts, and osteoclasts^12,13^. It is believed that inflammatory cytokines and parathyroid hormone-related protein secreted by breast cancer cells can stimulate osteoblasts to produce receptor activator of nuclear factor-κB (NF-κB) ligand (RANKL) and further enhance osteoclast differentiation and bone resorption^12,14^. Thus, a number of factors with potential chemoattractive properties are released to stimulate breast cancer cell proliferation and migration^15^. Bisphosphonate and denosumab have been shown to slow down the progression of breast cancer-induced osteolysis^16,17^. However, due to adverse events, such as osteonecrosis of the jaw, toothache, and hypocalcemia, and because antiresorptive treatment is only palliative, novel therapies for breast cancer-induced osteolysis should be considered.

The aim of this study was to assess the effects of RA on osteoclasts, osteoblasts, and MDA-MB-231 breast cancer cells. Subsequently, we evaluated the effects of RA in mouse models of Ti-particle-induced calvarial osteolysis and breast cancer-induced osteolysis. The related molecular mechanisms were further determined.

## Results

### RA inhibited RANKL-induced osteoclast formation in vitro

**Fig. 1:**
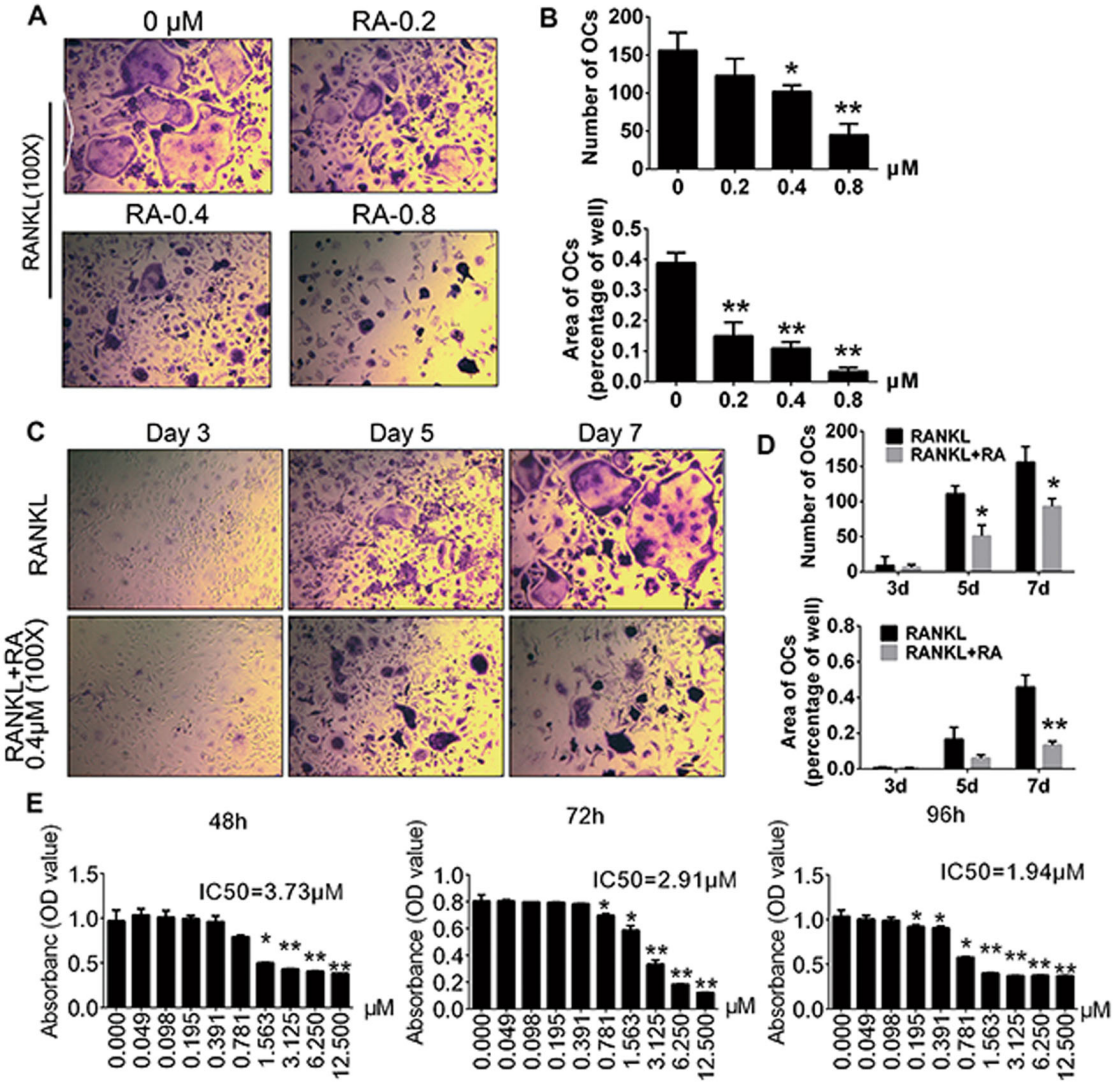
RA inhibited RANKL-induced osteoclastogenesis in vitro. **a** BMMs were cultured for 7 days with different concentrations of RA, M-CSF (30 ng/mL), and RANKL (50 ng/mL), and then subjected to TRAP staining (*n* = 3 per group). **b** The numbers and areas of osteoclasts from **a** were measured. **c** BMMs were treated with or without RA (0.4 μM) with the addition of M-CSF (30 ng/mL) and RANKL (50 ng/mL) for 3, 5, and 7 days and then subjected to TRAP staining (*n* = 3 per group). **d** The number and areas of osteoclasts from **c** were measured. **e** Cell viability of RA-treated BMMs tested by CCK-8 assays at 48, 72, and 96 h (*n* = 3 per group) (*p < 0.05, **p < 0.01)

To explore the effect of RA on RANKL-induced osteoclast differentiation, bone marrow-derived macrophages (BMMs) were treated with 0, 0.2, 0.4, and 0.8 µM RA in the presence of macrophage-colony stimulating factor (M-CSF) and RANKL. RANKL differentiated BMMs into mature tartrate-resistant acid phosphatase (TRAP)-positive multinucleated osteoclasts, but RA produced an inhibitory effect on the formation of TRAP-positive multinucleated osteoclasts in a concentration-dependent manner (Fig. 1a, b). We further treated BMMs with 0.4 µM RA for 3, 5, and 7 days. As shown in Fig. 1c, RA significantly suppressed osteoclast formation at day 7. The number of dead osteoclasts was also calculated and an increase of osteoclast apoptosis was observed with the increasing of the RA doses (Supplementary 1A, B). The results of cytotoxicity assays on BMMs revealed that slight cytotoxic effect was observed for a dose of 0.391 µM, and no significant inhibitory effects for doses below 0.195 µM (Fig. 1e). Collectively, these evidences suggested that RA prevented RANKL-induced osteoclast formation in vitro.

### RA suppressed RANKL-induced osteoclast-related gene expression in vitro

**Fig. 2:**
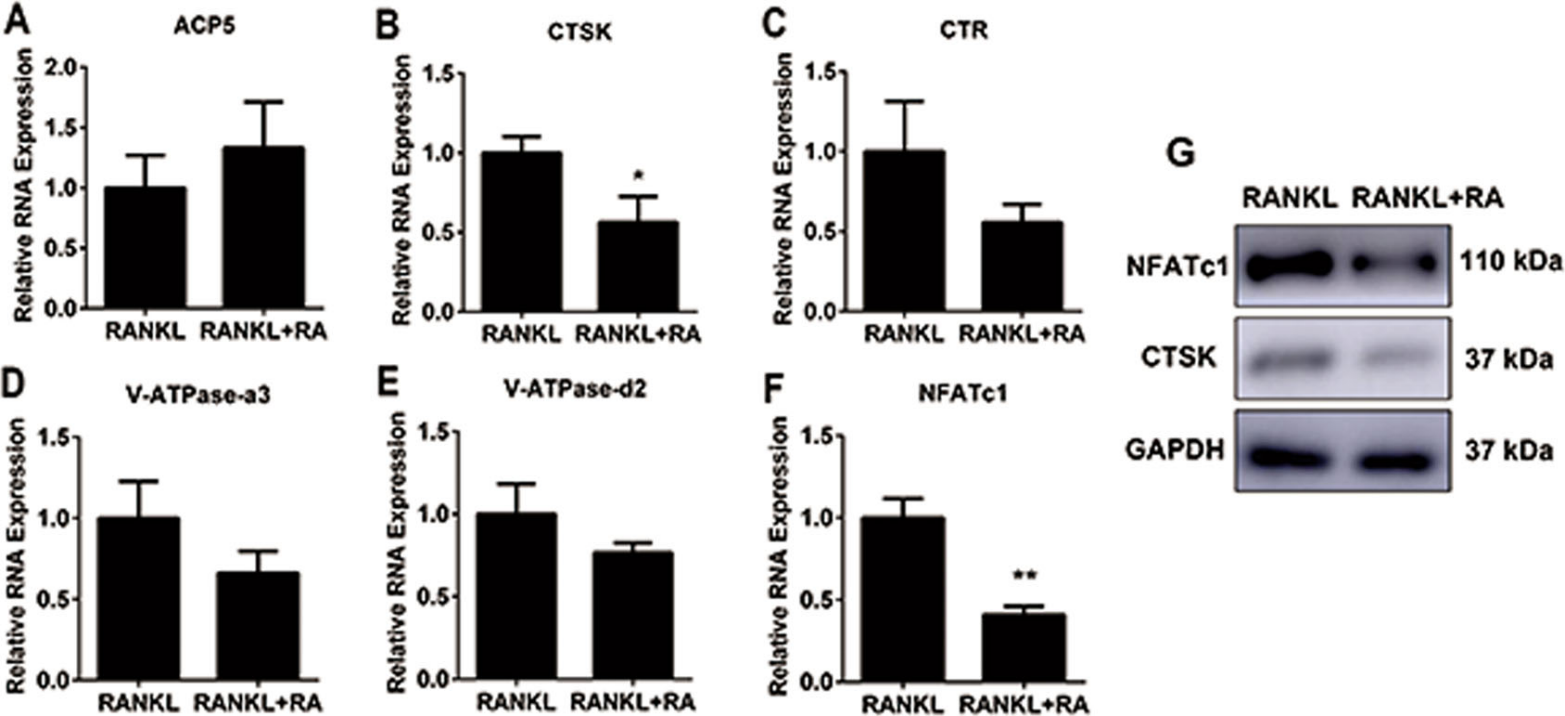
RA inhibited osteoclast-related gene expression in vitro. **a–f** Relative expression of osteoclast-specific genes (*ACP5*, *CTSK*, *CTR*, *V-ATPase-a3*, *V-ATPase-d2*, *NFATc1*) from 5-day RANKL-induced BMMs treated with RA (0.4 μM). The expression level was assessed by real-time PCR (*n* = 3 per group). **g** BMMs were treated with or without RA (0.4 μM) for 7 days. Western blotting for CTSK and NFATc1 was analyzed with the cell lysates (*p < 0.05, **p < 0.01)

To confirm the inhibitory potential of RA on RANKL-induced osteoclast differentiation, we examined the osteoclast-related genes, including TRAP (ACP5), cathepsin k (CTSK), calcitonin receptor (CTR), V-ATPase-a3, V-ATPase-d2, and the nuclear factor of activated T cells 1 (NFATc1). Compared to the control group treated by RANKL, the expression of CTSK and NFATc1 was dramatically suppressed with the addition of RA (Fig. 2a–f). The protein expression level of CTSK and NFATc1 was also decreased in the RA treatment group (Fig. 2g). These data confirmed that RA inhibited the expressions of osteoclast-related genes.

### RA inhibited osteoclastic bone resorption in a concentration-dependent manner

**Fig. 3:**
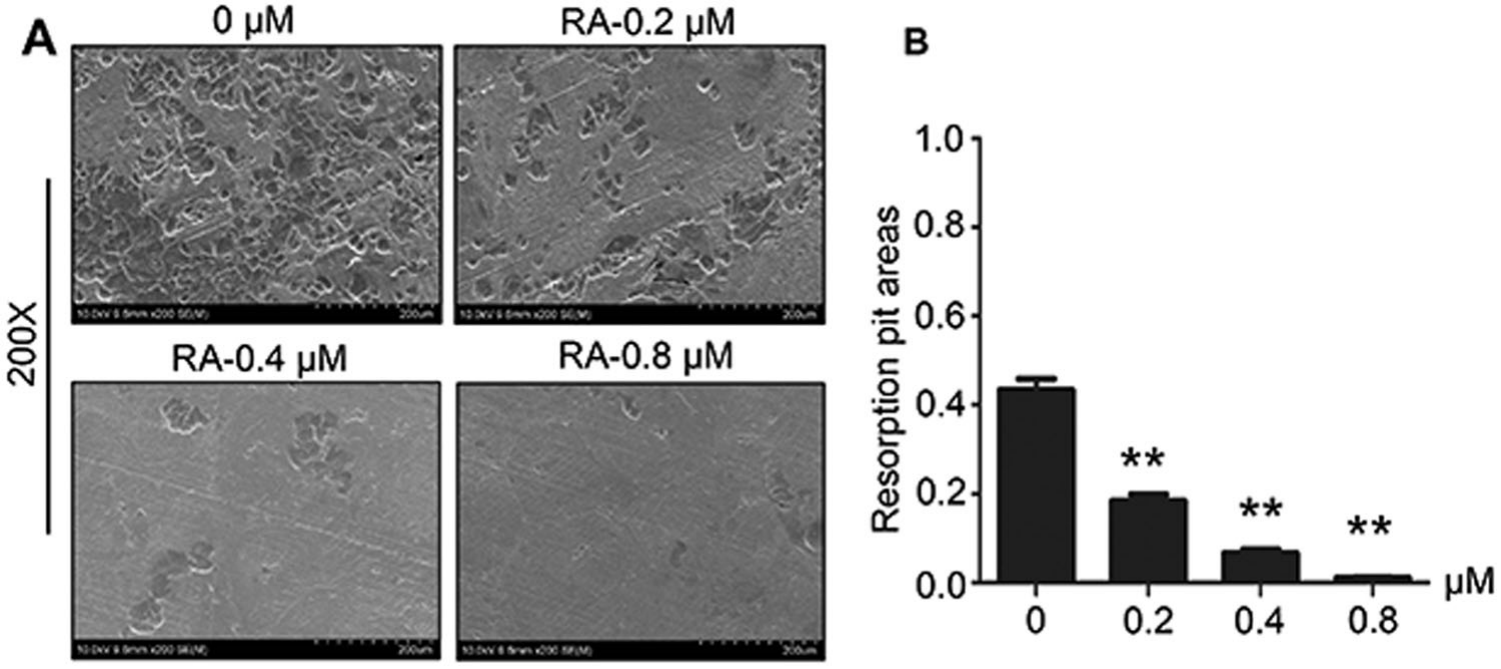
RA inhibited osteoclastic bone resorption activity in vitro. **a** BMMs were treated with various concentrations of RA, M-CSF (30 ng/mL), and RANKL (50 ng/mL) until mature osteoclasts formed and functioned. Bone resorption pits were shown by scanning electron microscope (SEM). **b** Resorption pit areas were quantified as mentioned in Materials and methods (*n* = 3 per group) (**p < 0.01)

We performed pit formation assay to investigate the function of RA on the osteoclastic bone resorption activity. BMMs without RA treatment obviously resorbed the bone surface (Fig. 3a), while the RA treatment group showed fewer resorption pits and almost no resorption pits were observed in the 0.8 µM RA group. The average resorption area in each group were 43, 18, 7, 1%, respectively (Fig. 3b). These results suggested that RA inhibited osteoclastic bone resorption in vitro.

### RA did not inhibited osteoblast differentiation and osteoblastic-related genes expression in vitro

No inhibitory effect was observed on the survival of MC3T3-E1 cells below doses of 0.781 µM (Supplementary 2A). To determine the role of RA on osteoblast differentiation, we further cultured MC3T3-E1 cells and analyzed alkaline phosphatase (ALP) activity at day 7. No significant difference of ALP was detected between control and the 0.2, 0.4, and 0.8 µM RA treatment groups (Supplementary 2B, D). We also evaluated osteoblastic mineralization with Alizarin red staining at day 21, and found that more total mineralized area was observed in 0.2 µM RA compared to the control group (Supplementary 2C, E). Though no significant difference between RA treatment and control group was observed in the mRNA expressions of osteoblastic-specific genes at day 7; however, secreted protein acidic and rich in cysteine were significantly increased after 14 days of treatment with RA (Supplementary 2F). The above results suggested that RA, at least, had no inhibitory effect on osteoblast differentiation.

### RA suppressed Ti-particle-induced osteolysis in vivo

**Fig. 4:**
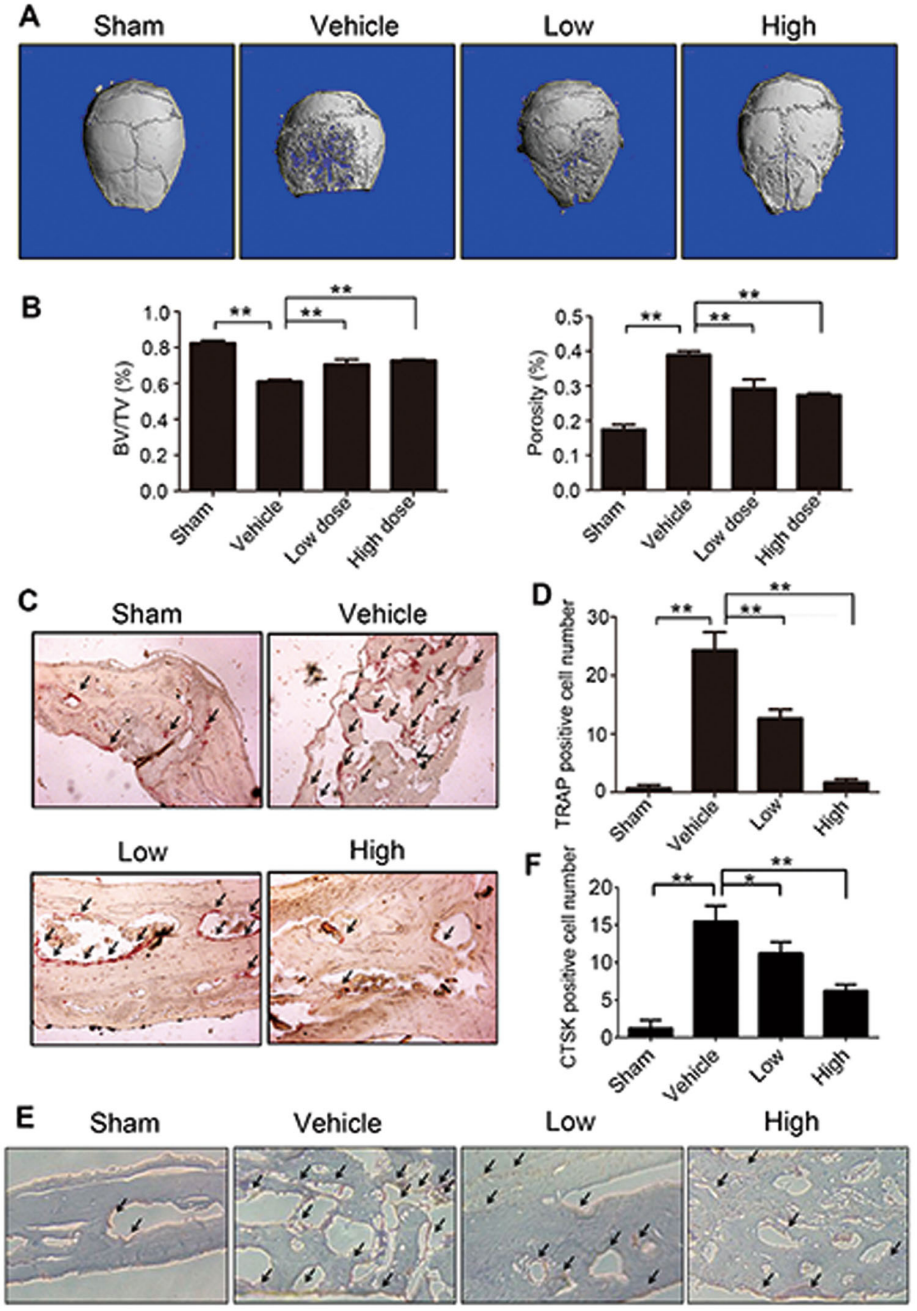
RA inhibited Ti-particle-induced mouse calvarial osteolysis in vivo. **a** Representative micro-CT and 3D reconstructed images from each group are shown (*n* = 6 per group). **b** The BV/TV and the percentage of total porosity of each group were measured. **c** TRAP staining was used to access RA prevention of titanium-particle-induced murine calvarial osteolysis. TRAP-positive osteoclasts were indicated by black arrows. **d** The number of TRAP-positive cells per field of tissue was determined. **e** CTSK staining was used to evaluate RA prevention of titanium-particle-induced murine calvarial osteolysis. CTSK-positive osteoclasts were indicated by black arrows. **f** The number of CTSK-positive cells per field of tissue was calculated (magnifications: ×100; *p < 0.05, **p < 0.01)

Since RA could inhibit osteoclastic bone resorption in vitro, we further explored its property on Ti-particle-induced osteolysis in a mouse calvarial model. Micro-computed tomography (CT) revealed that massive surface erosion was seen in the vehicle group. On the contrary, treatment with low or high concentration of RA significantly reduced Ti-particle-induced osteolysis (Fig. 4a). We then measured and calculated the ratio of bone volume to total volume (BV/TV) as well as the percentage of total porosity in the region of interest from three-dimensional (3D) reconstruction images. Compared with the vehicle group, treatment with low or high concentration of RA significantly increased the BV/TV and decreased the percentage of porosity (Fig. 4b). Meanwhile, TRAP staining indicated that the number of multinucleated osteoclasts (arrows) lining along the eroded bone surface was increased in the vehicle group, but was significantly decreased after low or high dose of RA treatment (Fig. 4c, d). CTSK immunohistochemical staining showed similar trends (Fig. 4e, f). These results illustrated that RA also inhibited osteoclasts formation and function in vivo.

### RA inhibited SRC/AKT signaling during osteoclastogenesis

**Fig. 5:**
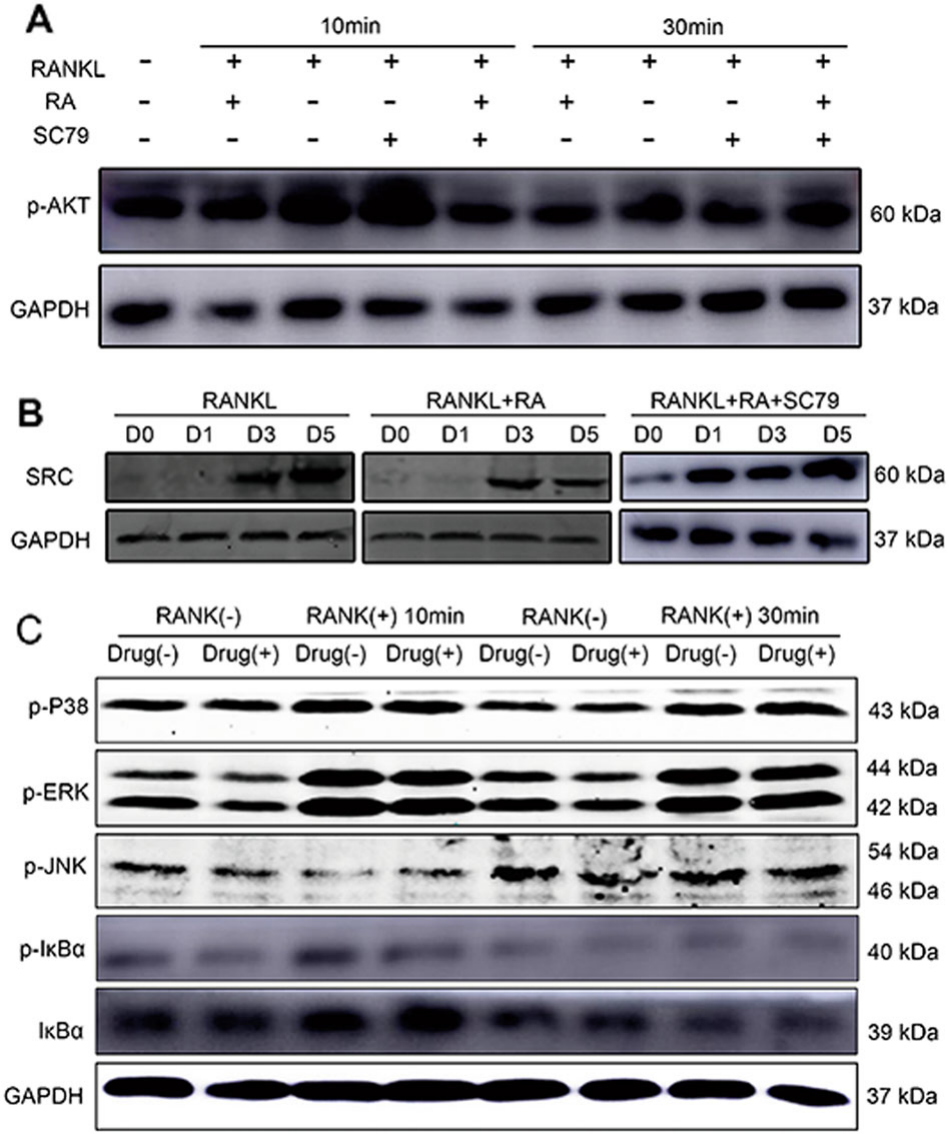
RA specifically inhibited SRC/AKT signaling pathways during osteoclastogenesis. **a** RAW264.7 cells were treated with or without RANKL (50 ng/mL), RA (0.8 μM), or SC79 (25 μM) for 0, 10, or 30 min, respectively. Western blotting for p-AKT was analyzed with the cell lysates. **b** RAW264.7 cells were treated with or without RANKL (50 ng/mL), RA (0.8 μM), or SC79 (25 μM) for 0, 1, 3, or 5 days respectively. Western blotting for SRC was analyzed with the cell lysates. **c** RAW264.7 cells were treated with or without RA (0.8 μM) with the addition of RANKL (50 ng/mL) for 0, 10 or 30 min, respectively. Western blotting for MAPK and IκBα signaling pathways was analyzed with the cell lysates

We next focused on elucidating the potential mechanism of RA in inhibiting osteoclasts formation and function. RAW264.7 cells were cultured with RANKL for different time to investigate mitogen-activated protein kinase (MAPK), NF-κB, and SRC/AKT signaling pathways. We found the RANKL-induced phosphorylation of AKT was significantly inhibited by RA at 10 and 30 min (Fig. 5a). This inhibitory effect can be partly rescued by the AKT activator, SC79. Moreover, expression of SRC increased since day 3 after stimulating by RANKL, but the addition of RA significantly inhibited this trend at the same time point (Fig. 5b). SC79 could reverse the RA-related decrease of SRC. However, RA did not show any suppressive effect on the RANKL-induced phosphorylation of c-Jun N-terminal kinase (JNK), p38, extracellular signalregulated kinase (ERK), as well as degradation of IκBα (Fig. 5c). These results revealed that RA specifically inhibited the SRC/AKT signaling pathways during osteoclastogenesis without affecting MAPK or NF-κB signaling pathways.

### RA inhibits breast cancer-associated osteolysis in vivo

**Fig. 6:**
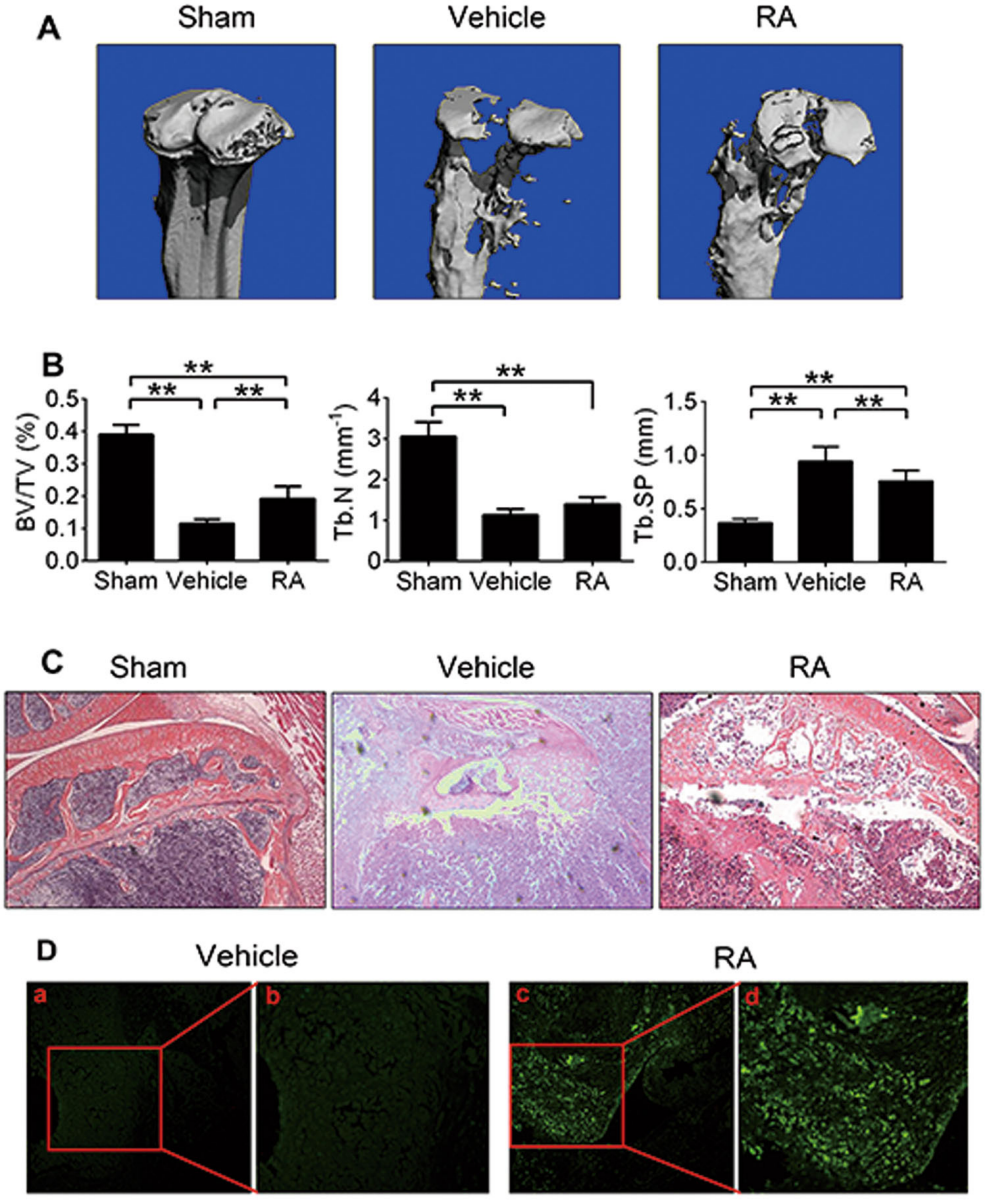
RA inhibits breast cancer bone metastasis and osteolysis in vivo. MDA-MB-231 cells were injected directly into the tibiae plateau. After 28 days of treatment, representative micro-CT and three-dimensional reconstructed images (**a**), quantitative data from micro-CT, including BV/TV, trabecular number (Tb.N), and trabecular separation (Tb.Sp) of each group (**b**) were acquired (*n* = 6 per group). Sections of tibiae were stained with H&E (**c**) and TUNEL (**d**). Magnifications: ×100 (**c**), and (a, c); and ×200 (b, d) (**p < 0.01)

To determine whether RA suppressed breast cancer-associated osteolysis, MDA-MB-231 cells were injected in mice tibiae plateau and treated with phosphate-buffered saline (PBS) or RA (100 µg/kg) for 28 days. Micro-CT and histology was performed to assess osteolytic bone metastasis. Compared with the RA treatment group, trabecular bone loss in the mice tibias was more remarkable in the vehicle group (Fig. 6a). Quantitative analysis revealed that the RA treatment group had significantly higher BV/TV ratios and smaller trabecular separation (Tb. Sp) compared to the vehicle group (Fig. 6b). From histology, extensive trabecular bone resorption and discrete cortical bone could be observed in the vehicle group, while intact bone cortex was remained in the RA treatment group (Fig. 6c). The transferase-mediated dUTP nick end labeling (TUNEL) assay further revealed that the degree of apoptosis was significantly increased in the RA treatment group compared to the vehicle group (Fig. 6d). All above data suggested that RA could inhibit breast cancer-induced osteolytic lesions.

### RA inhibits growth and invasion of breast cancer cells in vitro through promotion of apoptosis and inhibition and AKT/mTOR signaling

**Fig. 7:**
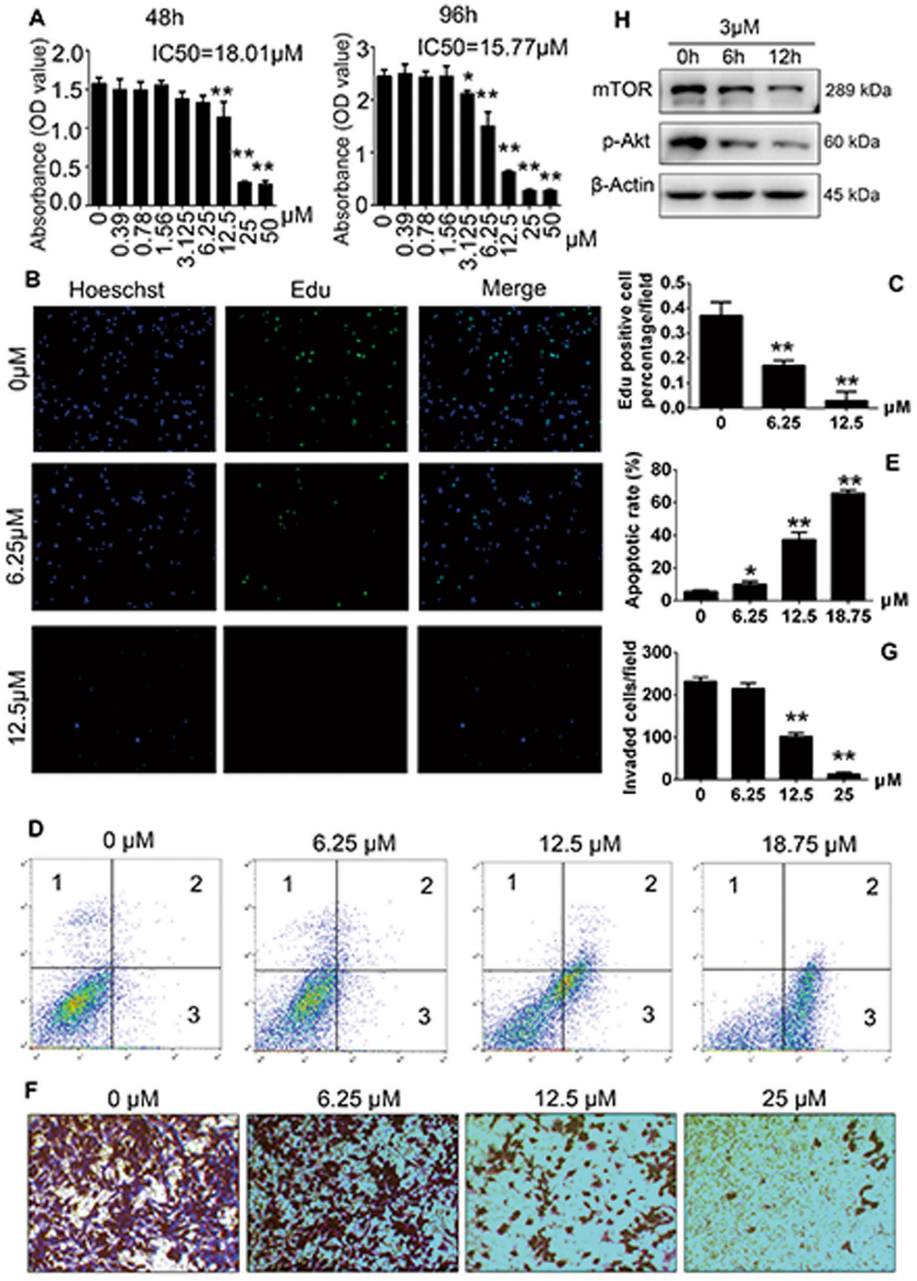
RA inhibits the proliferation and invasion of MDA-MB-231 cells through promotion of apoptosis and inhibition of AKT/mTOR signaling pathways. **a** Cell viability of RA-treated MDA-MB-231 cells tested by CCK-8 assays at 48 and 96 h. **b** MDA-MB-231 cells were treated with various concentrations of RA for 24 h and then evaluated with EdU incorporation assay (*n* = 3 per group). Magnifications: ×100. **c** The percentages of EdU-positive cells for each field. **d** MDA-MB-231 cells were treated with various doses of RA for 48 h and then stained with Annexin V and propidium iodide for flow cytometric analysis (*n* = 3 per group). **e** Apoptotic rate was defined as the percentage of dead and apoptotic cells (quandrants 2 and 3). **f** RA inhibited the invasion of MDA-MB-231 cells by Transwell invasion assay (*n* = 3 per group). Magnifications: ×200. **g** The number of invaded cells of each field was counted. **h** MDA-MB-231 cells were treated with or without RA (3 μM) for 0, 6, or 12 h, respectively. Western blotting for p-AKT and mTOR was analyzed with the cell lysates (*p < 0.05, **p < 0.01)

We further explored the mechanism how RA regulated the growth of breast cancer cells. Cell Counting Kit-8 (CCK-8) assays were performed on MDA-MB-231 cells after a 48 and 96 h culture, and RA treatment significantly decreased cell amount at doses higher than 6.25 μM (Fig. 7a). Next, we used ethynyl-2-deoxyuridine (EdU) incorporation assays to determine the effect of RA. After RA treatment for 24 h, the proliferation of MDA-MB-231 cells showed a significant decrease at both 6.25 and 12.5 μM doses (Fig. 7b, c). The flow cytometric analysis revealed that RA could increase the percentages of apoptotic cells (Fig. 7d, e). We used the transwell assay to examine the effect of RA on cell invasion. Our results revealed that RA significantly reduced the invasion of MDA-MB-231 cell in a concentration-dependent manner (Fig. 7f, g). We also used another breast cancer cell, BCAP37, and generated similar results (Supplementary 3). Further, MDA-MB-231 cells were cultured with RA for 0, 6, and 12 h to investigate AKT/mTOR signaling pathways. Both phosphorylation of p-AKT and expression of mTOR was significantly downregulated by treatment with RA (3 µM), indicating the inhibitory effect of RA on AKT/mTOR signaling (Fig. 7h). These data suggested RA could suppress the growth and invasion of breast cancer cells in vitro with inhibition of AKT/mTOR signaling.

## Discussion

One of the major causes of cancer-associated death among women is breast cancer, and bone is the major site of metastasis in invasive breast cancer^18^. The mechanisms underlying bone metastasis in breast cancer are still unclear; however, the concept of the “vicious cycle” during bone breakdown and tumor invasion has been widely accepted^19^. It is believed that pro-osteoclastic factors released by tumor cells stimulate osteoclastogenesis, whereas pro-tumorigenic growth factors secreted from the bone matrix promote tumor expansion^12–15^. Currently, no available treatment is sufficient to treat bone metastasis and resulting osteolysis^20^. RA is one such compound that is derived from anemone raddeana Regel and has been demonstrated to suppress the growth of gastric and colorectal tumors^6,9^. Our results revealed that RA possessed inhibitory effects on breast cancer-associated osteolysis through suppression of osteoclasts and breast cancer cells. The possible mechanisms might be that RA inhibited the SRC/AKT signaling pathway in osteoclasts as well as AKT/mTOR signaling in breast cancer cells.

Our study provided evidence for the effects of RA on RANKL-induced osteoclastogenesis. Different doses of RA were used in our experiment, and the number of TRAP-positive multinuclear osteoclasts was significantly decreased after RA exposure. The levels of osteoclast phenotypic markers, including CTSK and NFATc1, were also downregulated following the addition of RA. Furthermore, results of bone resorption assays indicated that the area of bone resorption pits was significantly reduced when treated with RA. The effects of RA on Ti-particle-induced osteolysis were further explored with a murine calvarial model. Micro-CT assessments demonstrated that Ti-particle-induced osteolysis was obviously inhibited in the RA treatment group compared with that in the control group.

To elucidate the molecular mechanisms underlying the above results, we first investigated the effects of RA on the RANKL-initiated signaling pathway, because RANKL has been shown to be a key regulator of osteoclast activation by breast cancer cells^12,14,21^. RANKL-induced signaling pathways include MAPK, NF-κB, and SRC/AKT pathways, which play a pivotal role in osteoclast differentiation and function^22–24^. A significant outcome of our study was that the RANKL-related SRC expression in osteoclasts was significantly downregulated after treatment with RA. Previous studies have shown that SRC is essential for the normal function of osteoclasts. Inhibition of SRC suppresses osteoclastogenesis and the formation of resorption pits^25^, which was consistent with our results. Although the numbers of osteoclasts increased compared with that in wild-type mice, Src^−/−^ mice developed osteopetrosis, suggesting the vital role of SRC in osteoclast function rather than differentiation^26^. AKT is a downstream target of SRC in response to RANKL^27^. TNF receptor-associated factor 6 (TRAF6) is recruited upon the activation of RANK by RANKL, which also leads to a complex of c-Src and TRAF6 and ultimately the activation of phosphoinositide 3-kinase (PI3K) and AKT^28,29^. Specifically, the expression of Src251, which lacks the entire kinase domain, inhibits AKT activity and osteoclast survival in transgenic mice^30^. In our study, decreased AKT phosphorylation was observed following the addition of RA, consistent with the above reports. We also found that the addition of AKT activator, SC79, can rescue the inhibitory effect of RA on AKT phosphorylation and SRC expression. Because MAPK and NF-κB signaling pathways were not affected by RA, it is tempting to speculate that RA may inhibit the formation and function of osteoclasts through downregulation of the SRC/AKT signaling pathway, which may explain why osteolysis was reduced in the RA group.

We then investigated the effects of RA on osteolysis using a breast cancer-associated osteolysis mouse model. Our results revealed that RA reversed severe osteolysis caused by MDA-MB-231 cells. Moreover, RA significantly increased BV/TV ratios and decreased trabecular separation compared with that in the vehicle group, which was in accordance with the results of histological analysis. In TUNEL assays, higher levels of apoptosis were detected in the RA treatment group than in the vehicle group.

Based on the above results, we further explored the direct effects of RA on MDA-MB-231 breast cancer cells. The survival and invasion of MDA-MB-231 cells was inhibited by RA, and RA also suppressed breast cancer cell proliferation and invasion. Flow cytometric analysis revealed that apoptosis rates in MDA-MB-231 cells increased significantly when treated with RA, in accordance with the results of TUNEL analysis. The mechanism may involve the inhibitions of phosphorylation of AKT and expression of mTOR. The PI3K/AKT/mTOR pathway is believed to be the main signaling pathway regulating cell proliferation, survival, metabolism, and angiogenesis^31–34^. Hyperactivation of the PI3K/AKT/mTOR pathway is frequently observed in breast cancer and is often associated with resistance to both anti-ERBB2-targeted and endocrine therapies^35^. Various PI3K/AKT/mTOR inhibitors have been identified as promising antitumor drugs in advanced breast cancer. Everolimus, an inhibitor of mTOR, was found to increase progression-free survival among patients in a phase 3, randomized trial^36^. Therefore, suppression of AKT activation and mTOR expression mediated the inhibitory effects of RA on breast cancer cell-associated osteolysis.

Another interesting finding in our study was that RA tended to promote osteoblast differentiation and osteoblastic-related genes expression in vitro. This is the first study reporting the potential effects of RA on osteoblast differentiation; however, further studies are still required to determine this effect and underlying mechanism.

In conclusion, RA exerted protective effects against breast cancer-associated bone osteolysis by decreasing osteoclast formation and resorption and by suppressing tumor cell proliferation and invasion. Analysis of the mechanisms involved in this process showed that RA inhibited SRC/AKT signaling in osteoclasts and AKT/mTOR signaling in MDA-MB-231 cells. Therefore, RA may serve as a potential therapeutic agent for the treatment of breast cancer-associated bone diseases in the future.

## Materials and methods

### Reagents and antibodies

RA was purchased from Meilunbio (Dalian, China). The alpha modification of Eagle’s medium (α-MEM), Dulbecco’s modified Eagle’s medium (DMEM), fetal bovine serum (FBS), and penicillin/streptomycin were obtained from Gibco-BRL (Gaithersburg, MD, USA). Recombinant mouse M-CSF and mouse RANKL were obtained from R&D (Minneapolis, MN, USA). SC79 was purchased from Selleck Chemicals (Texas, TX, USA). Specific antibodies targeting SRC, ERK, JNK, p38, IκBα, phospho-IκBα, phospho-ERK, phospho-JNK, phospho-p38, phospho-AKT, and glyceraldehyde 3-phosphate dehydrogenase (GAPDH) were obtained from Cell Signalling Technology (Cambridge, MA, USA). Antibodies against NFATc1 and CTSK were purchased from Abcam (Cambridge, MA, USA). CCK-8 was obtained from Dojindo Molecular Technology (Kumamoto, Japan). The TRAP staining kit, Triton X-100, and all other reagents were purchased from Sigma Aldrich (St. Louis, MO, USA), unless otherwise stated.

### Cell culture

BMMs were isolated from femoral and tibial bone marrow of 6-week-old female C57BL/6 mice, incubated in α-MEM containing 10% FBS, 100 U/mL penicillin/streptomycin, and 30 ng/mL M-CSF in a T75 flask in a 5% CO_2_ atmosphere at 37 °C until it reached 90% density. Then BMMs were moved to a 96-well plate at a density of 8 × 10^3^ cells per well and incubated for further differentiation. RAW264.7 cells and MC3T3-E1 cells were obtained from American Type Culture Collection. Human breast cancer cell lines (MDA-MB-231 and BCAP37) were gifts from Dr. Linbo Wang (Sir Run Run Shaw Hospital, Zhejiang University). They were cultured in DMEM supplemented with 10% FBS and antibiotics with the condition mentioned above. Cell culture media were replaced every 2 days.

### Cell viability assay

BMMs (8 × 10^3^ cells per well) were seeded into a 96-well plate, adherent cells were treated with various concentrations of RA in α-MEM containing 10% FBS, and 30 ng/mL M-CSF for 48, 72, or 96 h. MC3T3-E1 cells (5 × 10^3^ cells per well) were seeded into a 96-well plate with DMEM containing 10% FBS and treated with indicated concentrations of RA for 48 or 96 h. MDA-MB-231 cells (5 × 10^3^ cells per well) were seeded into a 96-well plate with DMEM containing 10% FBS and various concentrations of RA for 48 or 96 h. The culture medium was replaced every second days. The cytotoxic effect of RA on BMMs, MC3T3-E1, or MDA-MB-231 cells were assessed by a CCK-8 assay, 10 μL of CCK-8 buffer was added to each well, and plates were incubated for an additional 2 h. The absorbance was measured at 450 nm (650 nm reference) using an ELX800 microplate reader (Bio-Tek, USA).

### Bone resorption assay

BMMs (2 × 10^4^ cells per well) were seeded on bovine bone slices in 96-well plates for 24 h, and then stimulated with 0, 0.2, 0.4, and 0.8 µM RA in the presence of M-CSF (30 ng/mL) and RANKL (50 ng/mL) for another 3 days. Cells were then fixed with 2.5% glutaraldehyde. Bone slices were visualized under a scanning electron microscope (SEM, FEI Quanta 250; FEI, Hillsboro, OR, USA), and the resorption areas were quantified with Image J software (NIH).

### TRAP staining

BMMs were seeded into a 96-well plate at a density of 8 × 10^3^ cells per well and treated with 0, 0.2, 0.4, and 0.8 μM RA in the presence of 30 ng/mL M-CSF and 50 ng/mL RANKL. The culture medium was replaced every second day until mature osteoclasts were formed. Then, the cells were washed twice with PBS, fixed with 4% paraformaldehyde for 30 min, and stained for TRAP. TRAP-positive cells with five or more nuclei were counted under the light microscopy. To assess the survival of osteoclast, osteoclast ghosts were identified as dead osteoclasts and the total number of each well was calculated^37^.

### ALP and Alizarin red staining

MC3T3-E1were cultured into a 12-well plate and incubated with 0, 0.2, 0.4, or 0.8 µM RA in osteogenic medium (1 mM β-glycerophosphate and 5 μM l-ascorbic acid 2-phosphate). At day 7, ALP staining was performed and the area of positive cells was determined with Image J software (NIH). For Alizarin red staining, at day 21, cells were washed with PBS twice, fixed with 4% paraformaldehyde for 30 min, and stained with Alizarin red solution for 10 min at 4 °C. The area of Alizarin red S-stained mineralization nodules was also calculated with Image J software (NIH).

### EdU incorporation assay

Cell proliferation was evaluated with Click-iT EdU Cell Proliferation Kit (KeyGEN, Nanjing, China) following the manufacturer’s instructions. Breast cancer cells were pretreated with 0, 6.25, and 12.5 µM RA for 24 h. Then, the cells were incubated with 25 µM Edu for 2 h. Subsequently, the cells were fixed for 20 min with 4% paraformaldehyde. After permeabilization with 0.5% Triton X-100, the cells were incubated with 1× Click-iT EdU reaction cocktail for 30 min. Then, the cells were subjected to 1× Hoechst 33342 solution for 30 min. The cells were washed and observed under fluorescence microscopy.

### Flow cytometric analysis

Breast cancer cells were cultivated by the addition of 0, 6.25, 12.5, or 18.75 µM RA with medium described above for 24 h. Afterwards, the cells were washed twice with PBS and then resuspended in binding buffer. The cells were then stained with Annexin V and propidium iodide for 15 min at room temperature in the dark. Flow cytometric analyses were carried out using a flow cytometer, and the data were analyzed with the Cell Quest software, version 3.0 (BD Biosciences, Sunnyvale, CA, USA).

### Transwell invasion assay

A 24-well invasion chamber system was used to evaluate the effect of RA on invasion (Corning Inc., New York, NY, USA). Cells were seeded in the upper chamber at a density of 5 × 10^4^ cells in 200 µl serum-free medium by the addition of different concentrations of RA (0, 6.25, 12.5, and 25 µM). The lower chamber was filled with 500 μl of 10% fetal bovine serum-containing medium. The plates were incubated for 24 h at 37 °C. Then, the cells were fixed with methanol and stained with Trypan blue. Cotton swabs were used to remove the non-migrating cells on the upper side. The number of migrating cells was calculated by counting one randomly selected field of each well.

### Micro-CT assessment

The fixed calvaria and tibiae were analyzed by micro-CT scanner (Skyscan 1072; Skyscan, Aartselaar, Belgium). The scanning protocol was set at an isometric resolution of 9 mm, with X-ray energy settings of 80 kV and 800 μA. 3D images were reconstructed using Cone Beam Reconstruction software (SkyScan). BV, bone mineral density, BV/TV, mean trabecular number, and mean trabecular separation were recorded with resident reconstruction program (Skyscan).

### Histological analysis

After micro-CT analysis, the calvaria and tibiae were decalcified in 10% EDTA for 3 weeks, followed by paraffin embedding. Hematoxylin and eosin, TRAP, and CTSK staining were performed, after which specimens were examined and photographed under a high-quality microscope. The number of TRAP-positive and CTSK-positive multinucleated osteoclasts was counted.

### Deoxynucleotidyl TUNEL

Tumor tissues were decalcified in 10% EDTA for 3 weeks, and embedded in paraffin. TUNEL assay was performed with an In Situ Cell Death Detection Kit (Roche Applied Science, Indianapolis, IN, USA) according to the manufacturer’s instructions.

### Western blotting

Cells were lysed with RIPA buffer (Beyotime, Shanghai, China), then the lysate was centrifuged at 12,000 rpm for 10 min, and the protein in the supernatants was collected and quantified. Each protein lysate (30 µg) was resolved using sodium dodecyl sulfate–polyacrylamide gel electrophoresis and transferred to a polyvinylidene difluoride membrane (Millipore, Bedford, MA, USA). Following transfer, membranes were blocked with 5% skim milk for 2 h and probed with primary antibodies at 4 °C overnight and incubated with appropriate secondary antibodies. Antibody reactivity was detected by exposure in an Odyssey V3.0 image scanning (Li-COR Inc., Lincoln, NE, USA).

### RNA isolation and real-time PCR analysis

BMMs were cultured in 6-well plates at a density of 2 × 10^5^ cells per well, treated with 30 ng/mL M-CSF, 50 ng/mL RANKL, and 0, 0.2, 0.4, and 0.8 µM RA for 5 days. MC3T3-E1 cells were cultured in osteogenic medium at the same density with above indicated concentrations of RA for 7 or 14 days. Total RNA was extracted using the RNeasy Mini Kit (Qiagen, Valencia, CA, USA). RT-PCR was performed using SYBR Premix Ex Tag Kit (TaKaRa, Biotechnology, Otsu, Japan) and an ABI 7500 Sequencing Detection System (Applied Biosystems, Foster City, CA, USA). The following cycling conditions were used: denaturation at 95 °C for 10 min, 40 cycles at 95 °C for 10 s, and amplification at 60 °C for 34 s. The quantity of each target was normalized to GAPDH.

### Ti-particle-induced calvarial osteolysis mice model

A mouse calvarial osteolysis model was established using 8-week-old male C57BL/6 mice. After anesthesia, 30 mg of Ti particles were embedded under the periosteum at middle suture of calvaria in the Ti, low and high RA groups. In the sham group, the incision was closed without further intervention. Mice in the low or high RA groups were injected daily with 50 or 100 µg/kg per day RA, while mice in the sham or Ti group received PBS. After 14 days, mice were sacrificed and the calvariae were collected for micro-CT assessment and histological analysis.

### Breast cancer-induced osteolysis model

The model of human breast cancer bone metastasis was established through injection of the MDA-MB-231 cells (1 × 10^6^/mL) into the tibiae plateau of 5-week-old BALB/c nu/nu female mice. The mice were then randomly assigned to two groups, treated with PBS (*n* = 6) or RA (100 µg/kg body weight in vehicle, *n* = 6) by intraperitoneal injection every other day for 28 days and then sacrificed. The tibiae were scanned with a micro-CT and proceeded with histological or immunohistochemical analysis.

### Statistics analysis

The SPSS 20.0 software was used to analyze the data which were expressed as the mean ± SD. Groups were compared using the Student’s *t* test. Results with values of *P* < 0.05 were considered statistically significant.

### Ethical statement

All animal experiments were performed in accordance with guidelines for animal treatment of Sir Run Run Shaw Hospital. All experimental protocols in our study were approved by the Ethics Committee of Sir Run Run Shaw Hospital.

## Electronic supplementary material

Supplementary information

Supplementary 1

Supplementary 2

Supplementary 3
