# Supplementary material for: Raddeanin A suppresses breast cancer-associated osteolysis through inhibiting osteoclasts and breast cancer cells
Source: Cell Death Dis. 2018 Mar 7;9(3):376. doi: 10.1038/s41419-018-0417-0 (PMC5841366; doi:10.1038/s41419-018-0417-0)
Supplement: Supplementary file 1 — Supplementary information [file 41419_2018_417_MOESM1_ESM.docx]

**Supplementary 1. RA induced apoptosis of osteoclasts *in vitro*. (A)** BMMs were cultured for 7 days with different concentrations of RA, M-CSF (30 ng/mL) and RANKL (50 ng/mL) and then subjected to TRAP staining (n = 3 per group). Osteoclast ghosts were identified as dead osteoclasts (yellow arrow). **(B)** The number of dead osteoclasts of each well was calculated. (Magnifications: × 400; *p< 0.05, **p< 0.01).

**Supplementary 2.** **RA did not inhibited osteoblast differentiation and** **osteoblastic-related genes expression *in vitro*.** (**A**) Cell viability of RA-treated MC3T3-E1 cells tested by CCK8 assays at 48h and 96 h (n = 4 per group). **(B)** MC3T3-E1 cells were cultured for 7 days in differentiation medium with various concentrations of RA and then stained for Alkaline phosphatase (ALP). **(C)** BMSCs were cultured for 21 days in differentiation medium with various concentrations of RA and then stained for Alizarin Red. The ALP **(D)** and Alizarin Red **(E)** positive area of each well was quantified and expressed as a percentage of total well area. **(F)** MC3T3-E1 cells were cultured in differentiation medium with or without 0.4μM RA for 7 or 14 days. Relative expression of osteoblast-specific genes (RUNX2, Alpl, spp1, Bglap, collagen, sparc) was analyzed by real-time PCR. (*p< 0.05, **p< 0.01).

**Supplementary 3. RA inhibits the proliferation and invasion of BCAP37 cells through promotion of apoptosis and inhibition of AKT/mTOR signaling pathways.** (**A**) Cell viability of RA-treated BCAP37 cells tested by CCK8 assays at 48h and 96 h. (**B**) BCAP37 cells were treated with various concentrations of RA for 24h and then evaluated with EdU incorporation assay (n = 3 per group). Magnifications: × 100. (**C**) The percentages of EdU positive cells for each field. (**D**) BCAP37 cells were treated with various doses of RA for 48h and then stained with Annexin V and propidium iodide for flow cytometric analysis (n = 3 per group). **(E)** Apoptotic rate was defined as the percentage of dead and apoptotic cells (quandrants 2 and 3). **(F)** RA inhibited the invasion of BCAP37 cells by Transwell invasion assay (n = 3 per group). Magnifications: × 200. **(G)** The number of invaded cells of each field was counted. (*p< 0.05, **p< 0.01).
