# Supplementary figures and images for: Raddeanin A suppresses breast cancer-associated osteolysis through inhibiting osteoclasts and breast cancer cells
Source: Cell Death Dis. 2018 Mar 7;9(3):376. doi: 10.1038/s41419-018-0417-0 (PMC5841366; doi:10.1038/s41419-018-0417-0)

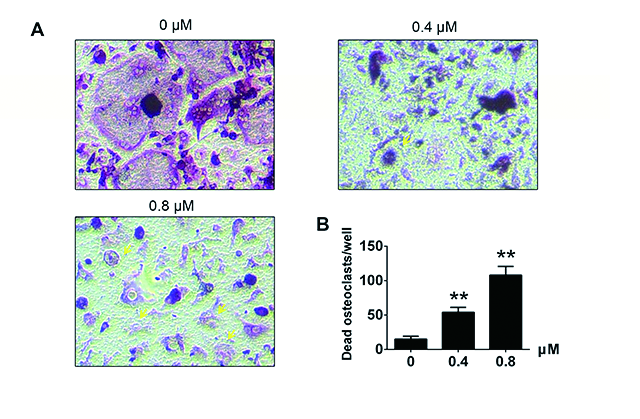

Supplement: Supplementary file 2 — Supplementary 1 [file 41419_2018_417_MOESM2_ESM.tif]

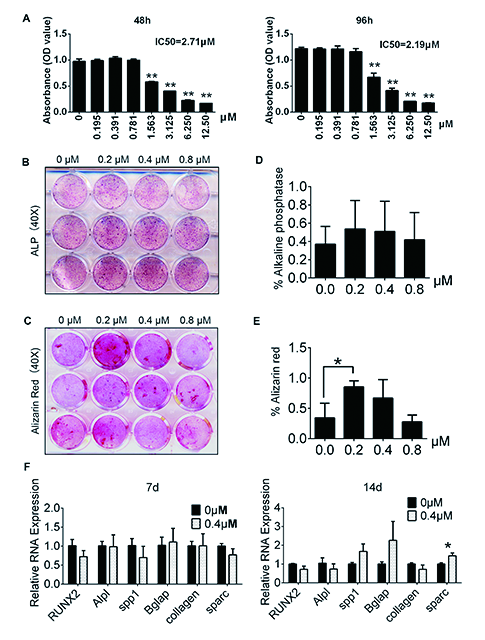

Supplement: Supplementary file 3 — Supplementary 2 [file 41419_2018_417_MOESM3_ESM.tif]

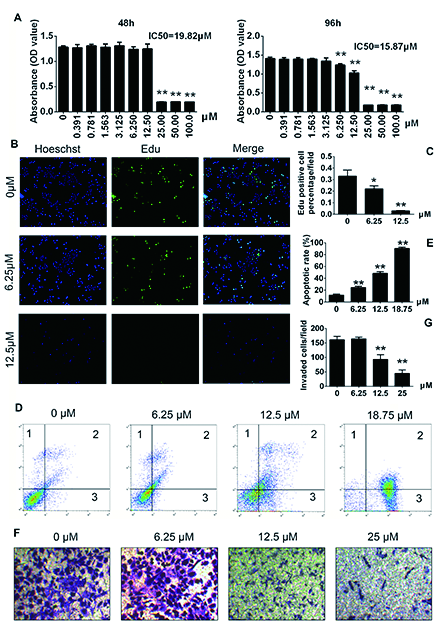

Supplement: Supplementary file 4 — Supplementary 3 [file 41419_2018_417_MOESM4_ESM.tif]
